# Supplementary material for: Deoxyribose and deoxysugar derivatives from photoprocessed astrophysical ice analogues and comparison to meteorites
Source: Nat Commun. 2018 Dec 18;9:5276. doi: 10.1038/s41467-018-07693-x (PMC6299135; doi:10.1038/s41467-018-07693-x)
Supplement: Supplementary file 1 — Supplementary Information [file 41467_2018_7693_MOESM1_ESM.pdf]

Supplementary Information

Deoxyribose and deoxysugar derivatives from photoprocessed astrophysical ice analogues and comparison to meteorites

Nuevo et al.

Supplementary Methods

Analysis of laboratory residues at room temperature. The three derivatization methods used in this work are described in detail below. With a few exceptions, these methods allowed for the detection of compounds containing 3 or more derivatizable OH groups. The molecular structures of the derivatives used in this study are shown in Supplementary Fig. 1. Note that the retention times for some compounds in the residues and their standards may be different, due to the fact that they were not necessarily injected the same day, in some cases several weeks apart. Also, injecting samples with higher concentrations (sometimes needed to identify compounds present in low abundances) may result in retention time shifts.

(+)-Butanol/TFAA derivatization. This method is a two-step process: (i) esterification of the carboxyl group of deoxysugar acids or formation of an acetal of the deoxysugars (or sugars) with an excess of a mixture of (+)-2-butanol ($C_4H_{10}O$; Norse Laboratories, high purity grade) and acetyl chloride (CH_3COCl; Fluka, ≥99.0%) ((+)-2-butanol:acetyl chloride = 10:0.5 v/v), followed by the heating of the mixture to 55°C for 45 min, followed by thorough drying; and (ii) reaction of the dried residues with an excess of a 3:4 mixture of trifluoroacetic anhydride (TFAA; $C_4F_6O_3$; Alfa Aesar, 99+%) and ethyl acetate ($CH_3COOC_2H_5$, anhydrous; Sigma-Aldrich, 99.8%), before heating to 55°C for 10 min. The latter step leads to the derivatization of all remaining hydroxyl groups into trifluoroacetyl esters (O-TFA). 1 µL of each derivatized sample is then injected in splitless mode into an Agilent 6890N gas chromatograph equipped with an Agilent J&W DB-17MS column (length: 60 m; diameter: 0.25 mm; film thickness: 0.25 µm), and coupled to an Agilent 5975 inert Mass-Selective Detector (QMS), with an injector temperature of 230°C, helium (UHP grade) as gas carrier with a flow of 1.0 mL min^{-1}, a transfer line temperature of 230°C, and an electron energy of 70 eV. Typical GC temperature program: start at 35°C, hold for 0.25 min, ramp to 70°C at 3°C min^{-1}, hold for 30 min, ramp to 90°C at 2°C min^{-1}, ramp to 230°C at 3°C min^{-1}, and hold for 60 min.

BSTFA derivatization. BSTFA-derivatized aliquots were prepared by mixing each dried sample with *N*,*O*-bis(trimethylsilyl)trifluoroacetamide (BSTFA; with 1% trimethylchlorosilane; Restek) in a 2:1 ratio, and by heating the mixture to 80°C for 1 hour to convert all compounds into their trimethylsilyl (TMS) derivatives. 1 µL of each BSTFA-derivatized aliquot was injected in splitless mode into a Thermo Trace gas chromatograph coupled to a DSQ II quadrupole mass spectrometer (QMS) equipped with an Agilent J&W DB-17HT column (30 m × 0.250 mm × 0.15 µm), with an injector temperature of 250°C, a helium flow of 1.3 mL min^{-1}, a transfer line at 250°C, an electron energy of 70 eV, and a temperature program as follows: start at 50°C, hold for 2 min, ramp to 250°C at 5°C min^{-1}, hold for 5 min.

MTBSTFA derivatization. Residues were derivatized with *N*-*tert*-butyldimethylsilyl-*N*-methyltrifluoroacetamide (MTBSTFA; with 1% *tert*-butyldimethylchlorosilane; Sigma-Aldrich) by adding 50 µL of ethyl acetate and 100 µL of MTBSTFA to the dried aliquots. Samples were then heated to 55°C for 30–60 min (generally with no evaporation or concentration), leading to the derivatization of the compounds into their *tert*-butyldimethylsilyl (*t*-BDMS) derivatives. 1 µL of each derivatized sample was then injected in splitless mode into an Agilent 6890N/5975 GC-MS device (column: Agilent J&W DB-17MS), with the following temperature program: start at 35°C, hold for 0.25 min, ramp to 250°C at 3°C min^{-1}, and hold for 70 min.

Supplementary Figures

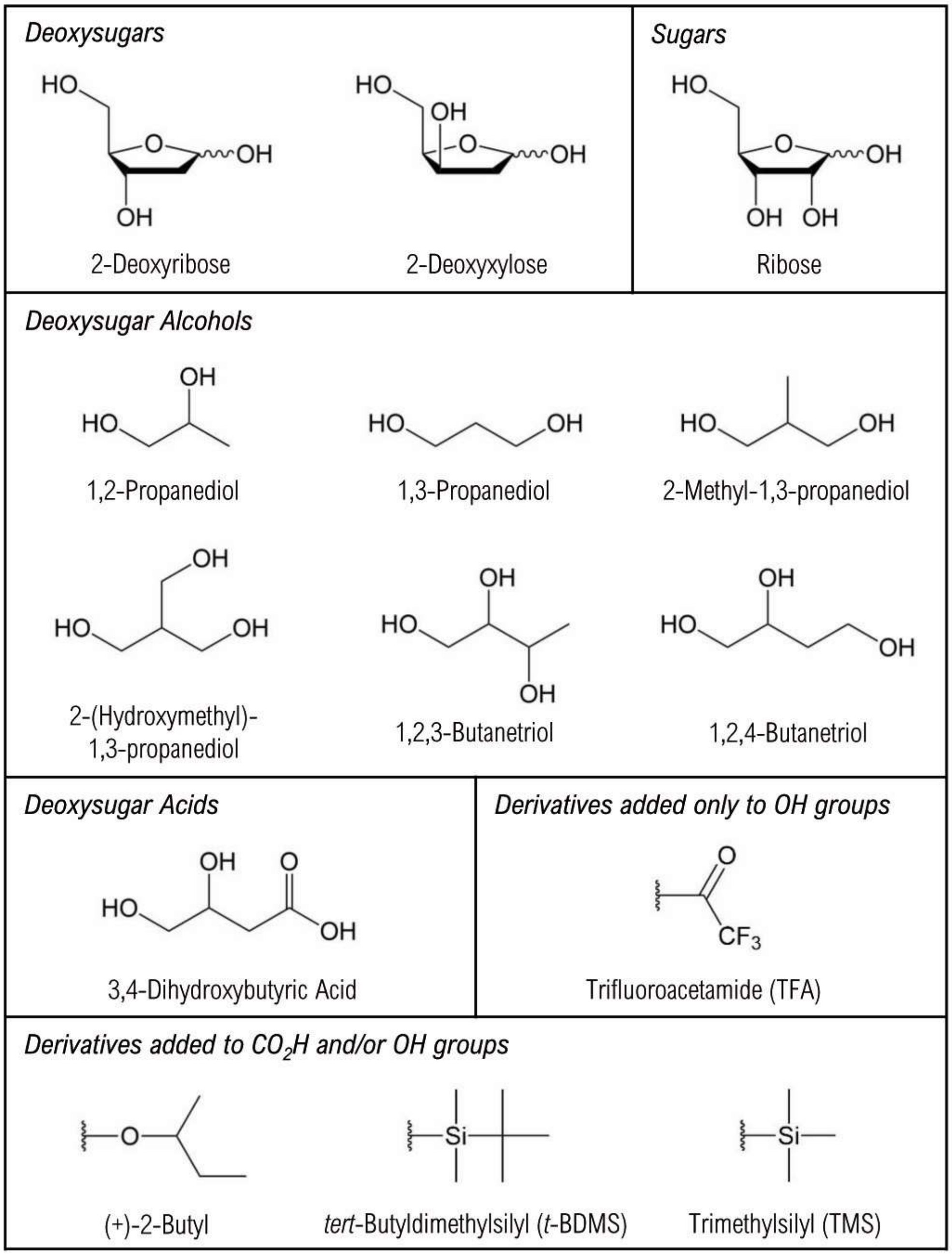

Supplementary Fig. 1 Molecular structures of all the compounds identified or tentatively identified in our residues (Table 1, Figs. 1, 2, and 3, and Supplementary Figs. 2, 3, and 4) and meteorites (Table 1 and Fig. 4), as well as the derivatives used in this study.

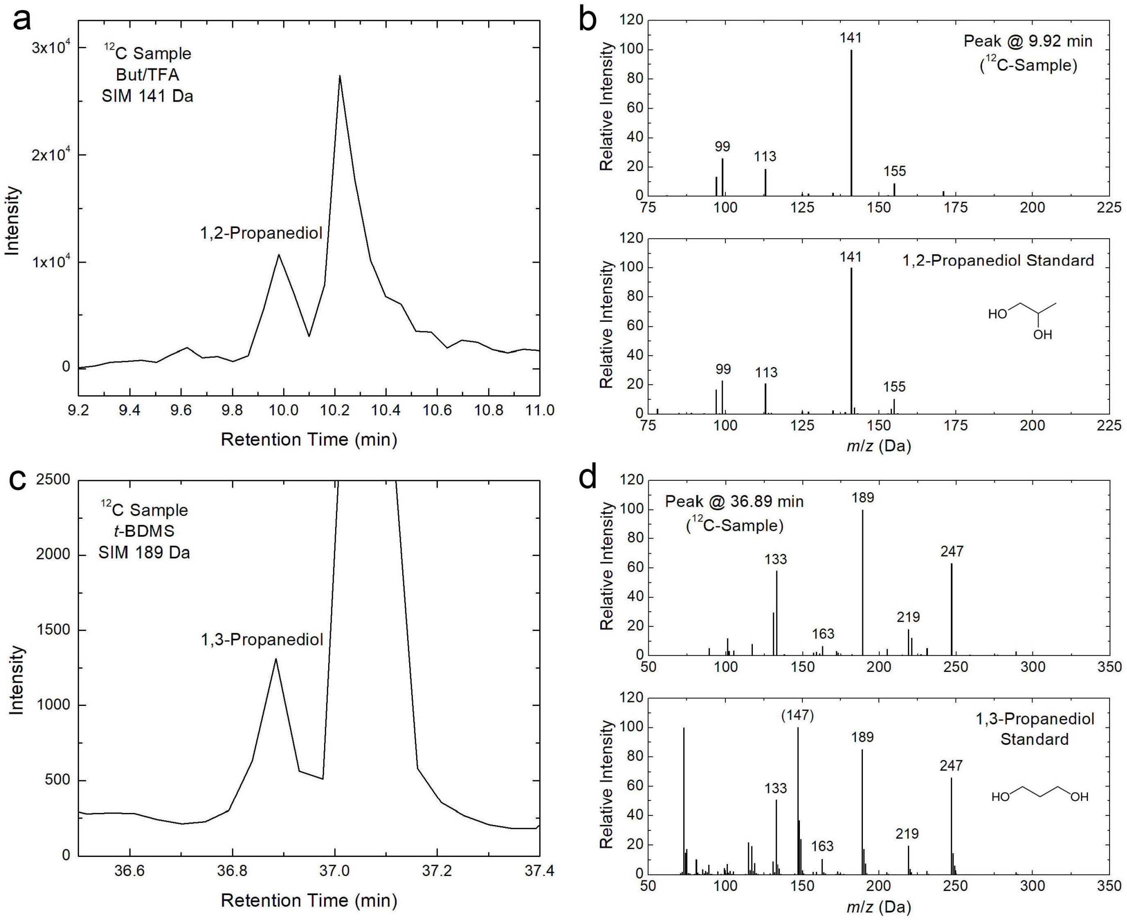

Supplementary Fig. 2 Identification of the 3C deoxysugar alcohols 1,2-propanediol and 1,3-propanediol in ice photolysis residues. **a**, Single-ion monitoring (SIM) chromatogram of a residue produced from the UV irradiation of an H_2O:CH_3OH (2:1) ice mixture (*m*/*z* = 141 Da) after derivatization with (+)-2-butanol/TFAA (the present deoxysugar alcohols only react with TFAA). **b**, Mass spectrum of the peak assigned to 1,2-propanediol in the same residue, compared with the mass spectrum of a standard of 1,2-propanediol. **c**, SIM chromatogram of a residue produced from the UV irradiation of a similar H_2O:CH_3OH (2:1) ice mixture (189 Da) derivatized with MTBSTFA. **d**, Mass spectrum of the peak assigned to 1,3-propanediol in the same residue, compared with the mass spectrum of a standard of 1,3-propanediol. Molecular structures are shown without derivatization. Assignments of the fragments in the mass spectra can be found in Supplementary Table 1.

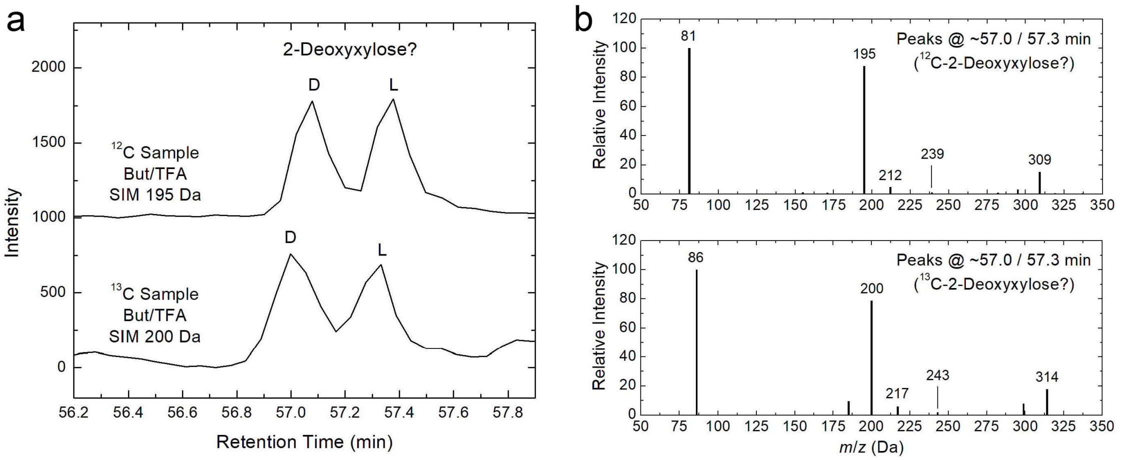

Supplementary Fig. 3 Tentative identification of 2-deoxyxylose in ice photolysis residues. **a**, SIM chromatograms of residues produced from the UV irradiation of H_2O:CH_3OH (2:1) (^{12}C sample, *m/z* = 195 Da) and H_2O:$^{13}CH_3OH$ (2:1) (^{13}C sample, 200 Da) ice mixtures after derivatization with (+)-2-butanol/TFAA, showing the peaks tentatively associated with the two enantiomers of 2-deoxyxylose. The tentative assignments of the D and L enantiomers are based on the presence of a corresponding low-abundance isomer of 2-deoxyribose in the respective D and L commercial standards of 2-deoxyribose. The isomers, that we assign to the D and L enantiomers of 2-deoxyxylose, have nearly identical mass spectra to that of 2-deoxyribose and believed to be a side product of the synthesis of 2-deoxyribose. Intensities are offset for clarity. **b**, Mass spectra of one peak from the chromatograms of the regular and ^{13}C-labelled residues. The mass spectra are very similar to those of ^{12}C- and ^{13}C-2-deoxyribose (Fig. 1b), and consistent with the fact that 2-deoxyxylose is the only possible diastereomer of 2-deoxyribose. Assignments of the fragments in the mass spectra are similar to those for 2-deoxyribose and can be found in Supplementary Table 1.

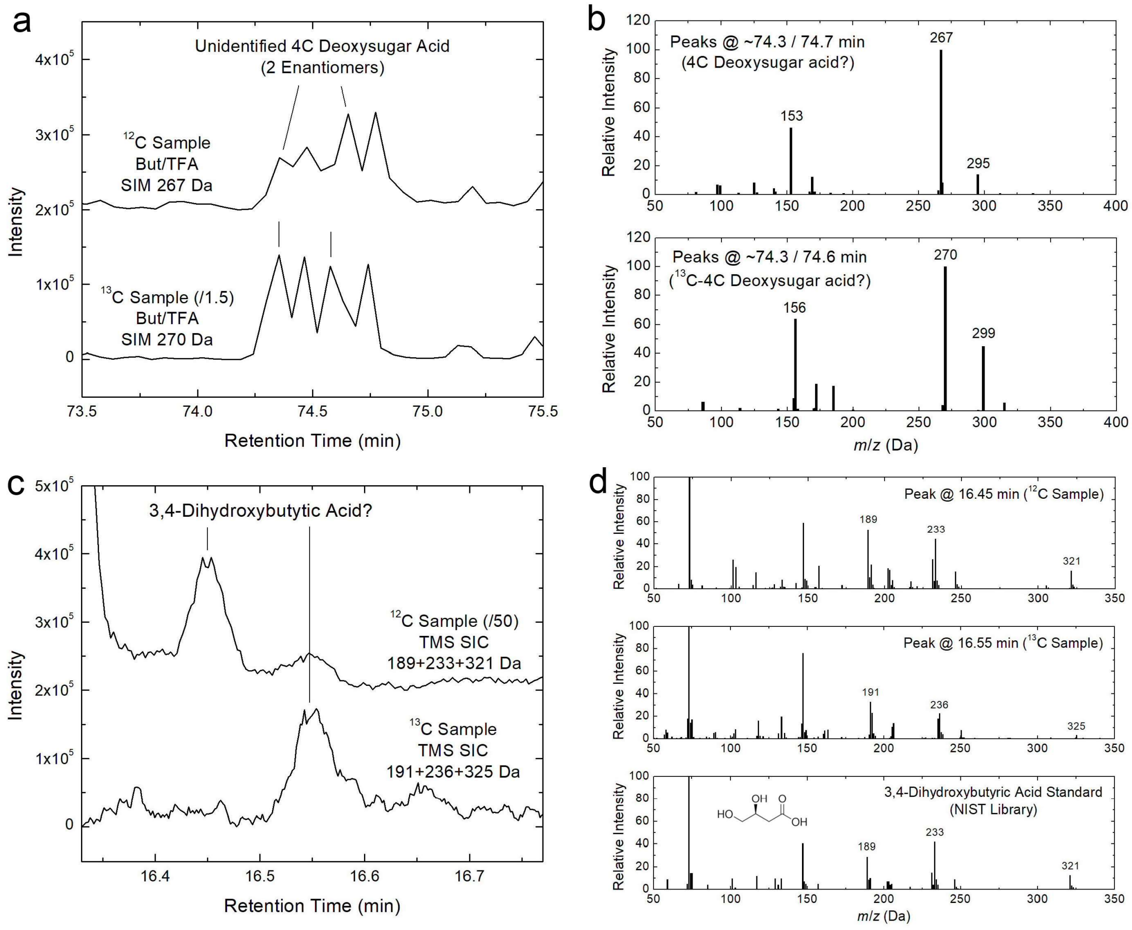

Supplementary Fig. 4 Peaks indicating the probable presence of both enantiomers of a 4C deoxysugar acid in the chromatograms of ice photolysis residues, most likely in its dimeric or higher oligomeric form. **a**, SIM chromatograms of residues produced from the UV irradiation of H_2O:CH_3OH (2:1) (^{12}C sample, *m/z* = 267 Da) and H_2O:$^{13}CH_3OH$ (2:1) (^{13}C sample, 270 Da) ice mixtures after derivatization with (+)-2-butanol/TFAA, showing the peaks associated with the two enantiomers of the deoxysugar acid, probably in its dimeric or a higher oligomeric form, as suggested by their relatively high retention times (~74–75 min) and the presence in their mass spectra of a fragment ion at 267 Da (270 Da for ^{13}C-labelled compounds). Subsequent analysis (not shown) of (+)-2-butyl/TFA derivatives of 4C deoxysugar acids has shown that this fragment is due to the loss of a carboxyl/butyl group. For reference, the monomers of (+)-2-butyl/TFA derivatives of 2,3-dihydroxybutyric acid elute at ~15–20 min. Intensities are offset for clarity. **b**, Mass spectra of one peak from the chromatograms of the regular and ^{13}C-labelled residues. Both peaks of a given chromatogram show the same mass spectra. Mass shifts (*bottom panel*) are consistent with a ^{13}C-labelled compound similar to a 4C deoxysugar acid. **c**, Single-ion chromatograms (SICs) of two similar residues produced from the UV irradiation of H_2O:CH_3OH (2:1) (189+233+321 Da) and H_2O:$^{13}CH_3OH$ (2:1) (191+236+325 Da) ice mixtures, showing one peak of interest. Intensities are offset for clarity. **d**, From top to bottom, mass spectra of these peaks from the chromatograms of the regular residue, the ^{13}C-labelled residue, and a TMS-derivatized 3,4-dihydroxybutyric acid from the NIST library. 3,4-Dihydroxybutyric acid was found in Murchison and Murray[1,2] (Table 1). The molecular structure of 3,4-dihydroxybutyric acid is shown without derivatization. Assignments of the fragments in the mass spectra can be found in Supplementary Table 1.

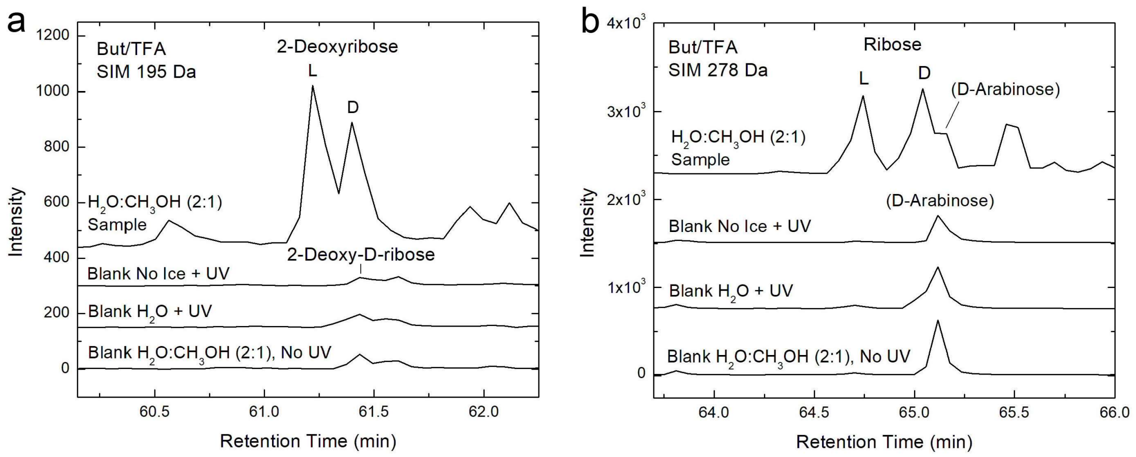

Supplementary Fig. 5 Determination of the level of contaminant 2-deoxyribose and ribose in the residues. **a**, From top to bottom, SIM chromatograms (*m/z* = 195 Da) of a residue produced from an H_2O:CH_3OH (2:1) ice mixture, a piece of Al foil with no deposited ices that was exposed to UV irradiation, a pure H_2O ice (no methanol) deposited and exposed to UV irradiation, and an H_2O:$^{13}CH_3OH$ (2:1) ice mixture deposited but UV irradiated, showing the peaks for the L and D enantiomers of 2-deoxyribose. **b**, From top to bottom, SIM chromatograms (278 Da) of the same residue and the same three control samples, showing the peaks for the L and D enantiomers of ribose. All chromatograms are plotted using the same intensity scale, with intensities offset for clarity. Chromatograms of control samples show some contaminant 2-deoxyribose in the control samples, but no contaminant ribose. Abundances of 2-deoxyribose in Tables 1 and 2 were corrected by subtracting the contribution of the contaminant 2-deoxyribose in the control sample in which an H_2O:$^{13}CH_3OH$ (2:1) ice mixture was deposited but not UV irradiated.

Supplementary Table 1 Molecular ions and fragments from the mass spectra of the compounds identified in residues and meteorites

Compounds	Underivatized Formulas (Masses in Da)	Derivatized Masses (Da) (^{12}C)	Derivatized Masses (Da) (^{13}C)	Structures of Molecular Ions and Fragments
1,2-Propanediol[a,†]	$C_3H_8O_2$ (76)	268*	(271)[†]	M^+ [2 × TFA]
		155	(158)[†]	M^+ – (TFA–O)
		141	(143)[†]	M^+ – (TFA–O–CH_2)
1,3-Propanediol[b,‡]	$C_3H_8O_2$ (76)	304*	(307)[‡]	M^+ [2 × *t*-BDMS]
		247	(250)[‡]	M^+ – (*t*-butyl)
		219	(221)[‡]	M^+ – (*t*-butyl) – (CO)
2-Methyl-1,3-propanediol[b]	$C_4H_{10}O_2$ (90)	318*	322*	M^+ [2 × *t*-BDMS]
		261	264	M^+ – (*t*-butyl)
2-(Hydroxymethyl)-1,3-propanediol[a,†]	$C_4H_{10}O_3$ (106)	394*	(398)[†]	M^+ [3 × TFA]
		281	(285)[†]	M^+ – (TFA–O)
		167	(171)[†]	M^+ – (TFA–O) – (TFA–OH)
		166	(170)[†]	M^+ – 2 (TFA–OH)
1,2,3-Butanetriol[a]	$C_4H_{10}O_3$ (106)	394*	398*	M^+ [3 × TFA]
		281	285	M^+ – (TFA–O)
		253	255	M^+ – (TFA–O–CH–CH_3)
		167	171	M^+ – (TFA–O) – (TFA–OH)
		141	143	TFA–O–CH–CH_3
1,2,4-Butanetriol[a]	$C_4H_{10}O_3$ (106)	394*	398*	M^+ [3 × TFA]
		281	285	M^+ – (TFA–O)
		267	270	M^+ – (TFA–O–CH_2)
		167	171	M^+ – (TFA–O) – (TFA–OH)
		166	170	M^+ – 2 (TFA–OH)
		153	156	M^+ – (TFA–O–CH_2) – (TFA–OH)
2-Deoxyribose[a], 2-deoxyxylose[a]	$C_5H_{10}O_4$ (134)	382*	387*	M^+ [butyl + 2 × TFA]
		309	314	M^+ – (butyl–O)
		195	200	M^+ – (butyl–O) – (TFA–OH)
		81	86	M^+ – (butyl–O) – 2 (TFA–OH)
Ribose[a]	$C_5H_{10}O_5$ (150)	494*	499*	M^+ [butyl + 3 × TFA]
		421	426	M^+ – (butyl–O)
		278	282	M^+ – (butyl–O) – (TFA–OH) – (CHO)
		193	198	M^+ – (butyl–O) – 2 (TFA–OH)

[a] Derivatized with (+)-2-butanol/TFAA; [b] Derivatized with MTBSTFA

[†] Only found in meteorites (Fig. 4), so there is no ^{13}C-labelled mass spectrum; [‡] Only detected in regular (^{12}C) residues (Supplementary Fig. 1)

*M^+, molecular ion (not observed); butyl, (+)-butyl group ($CH(CH_3)CH_2CH_3$, from derivatization; 57 Da); TFA, trifluoroacetyl group (CF_3–C=O, from derivatization; 97 Da); *t*-BDMS, *tert*-butyldimethylsylil group ($Si(CH_3)_2C(CH_3)_3$, from derivatization; adds 114 Da after loss of a hydrogen); *t*-butyl, *tert*-butyl group ($C(CH_3)_3$, from derivatization; 57 Da); CO, carbon and oxygen attached to a *t*-butyl group (28 Da for ^{12}C, 29 Da for ^{13}C); CHO, aldehyde group (29 Da for ^{12}C, 30 Da for ^{13}C)

The molecular structures of the derivatization tags are shown in Supplementary Fig. 1

Supplementary References

1. Cooper, G. *et al.* Carbonaceous meteorites as a source of sugar-related organic compounds for the early Earth. *Nature* **414**, 879–883 (2001).
2. Cooper, G. & Rios, A. C. Enantiomer excesses of rare and common sugar derivatives in carbonaceous meteorites. *Proc. Natl. Acad. Sci. USA* **113**, E3322–E3331 (2016).
